# Moonshots and metastatic disease: the need for a multi-faceted approach when studying atypical responses

**DOI:** 10.1038/s41523-017-0010-1

**Published:** 2017-03-16

**Authors:** Kristine De La Torre, Elly Cohen, Anne Loeser, Marc Hurlbert, on behalf of the Metastatic Breast Cancer Alliance

**Affiliations:** 1 Metastatic Breast Cancer Alliance, New York, NY USA; 2 BreastCancerTrials.org, San Francisco, CA USA; 3 Breast Cancer Research Foundation, New York, NY USA

## Abstract

Clinical research generally focuses on results involving a statistical mean with little attention in trial design to patients who respond considerably better or worse than average. Exploring the reasons underlying an “atypical response” will increase understanding of the mechanisms involved in cancer progression and treatment resistance, accelerate biomarker identification, and improve precision medicine by allowing clinicians to prospectively select optimal treatments. Based on our review, we suggest two ways to move this field forward. First, we suggest that clear categorization of “atypical responders” is needed. This encompasses three sub-categories of patients: “exceptional responders” (those with an unusually favorable treatment response), “rapid progressors” (patients demonstrating an unusually poor or no therapeutic response), and “exceptional survivors” (patients who have far outlived their initial prognosis). Such categorization may depend upon the clinical context and disease subtype. Second, we suggest that atypical responses may be due not only to somatic mutations in tumors, but also to inherited polymorphisms in non-tumor tissue, host and tumor environments, lifestyle factors, co-morbidities, use of complementary and integrative medicine, and the interaction among these components. Here, we summarize new research initiatives exploring atypical responses, the potential reasons for atypical responses, and a strategic call to action. Rigorous studies of normal and atypical responses to treatment will be needed to strengthen understanding of the role of non-tumor factors. Clinical trial design for targeted and other types of therapies should be enhanced to collect data in a standardized manner beyond tumor genetics, resulting in more thorough study of the whole patient.

## Introduction

The National Cancer Institute (NCI) states “Precision medicine uses the genetics of disease to identify effective therapies.”^1^ We suggest that a new era of oncological precision medicine will allow expansion of this definition and a paradigm shift away from treating only the disease (killing cancer cells) and will encompass treating the whole patient, considering not only physical health and genetics, but emotional well-being, lifestyle, and environmental factors during and after treatment. Precision medicine focuses on an individual patient and his or her unique characteristics. Identifying reasons for an atypical response will better guide precision medicine and inform clinical decisions. Studying patients who exhibit an atypical response will likely provide mechanistic insight into the phenomenon, identify novel biomarkers that can be used to prospectively identify patients who will^2–8^ and will not^2^ benefit from a particular therapy, and lead to novel combination therapies.^2, 4, 9^ We propose that 1. clear categorization of atypical responses is needed, and 2. atypical responses may be due to not only tumor genomic factors but also characteristics of the environment in which the tumor is located, external influences including a patient’s lifestyle choices and psychosocial support, and interactions among various factors. Emerging data regarding the influence of these components on response to therapy and disease progression are preliminary and not part of current standard of care, but are sufficiently compelling to require further study.

### Categories of atypical responders and the reasons for these outcomes

Some groups have suggested terminology for patients exhibiting atypical responses. The term “exceptional responders” has been used in clinical studies (Table 1). The NCI defined exceptional responders as part of its exceptional responders initiative.^10^ The AURORA clinical trial defines both unusually favorable and unusually poor responders, using the terms “exceptional responders” and “rapid progressors,” respectively.^11^ The Broad Institute’s Metastatic Breast Cancer Project encompasses all patients with metastatic breast cancer (MBC), including exceptional responders.^12^

**Table 1:** Current initiatives and published studies examining an atypical response

Study or Institute	Qualitative definition	Metrics/Quantitative criteria
NCI	“Exceptional responders” are patients who have a unique response to treatments that are not effective for most other patients.^10^	•Complete response to treatment expected in ≤10% of patients
		•Partial response to treatment >6 months expected in ≤10% of patients
		•Response at least three times the duration expected when therapy started
AURORA trial	Defines “exceptional responders” as those “showing (nearly) complete response for a duration exceeding 1 year”, and “rapid progressors” as “patients on first- or second-line treatment progressing within the first 3 months since its initiation”.^11^ A subset of AURORA trial patients will be considered atypical responders.	•Complete response for a duration of ≥1 year
		•Defines “rapid progressors”
		•Progressing in <3 months since initiation of 1st or 2nd line of therapy
MBC project	Uses the term “extraordinary responders” and “exceptional responders” .^12^	Initial definitions at the launch of the study include:
		For patients with ER+/HER2− disease or HER2+ MBC:
		•Duration with metastatic disease (overall survival (OS)), >10 years, OR
		•Duration on any one therapy (progression-free survival (PFS)), >3 years, OR
		•Any exceptional response to therapy (complete or near complete response), as determined by the investigators after review of the answers to the screening questions, OR
		•Any other clinical scenario that the investigators believe constitutes an extraordinary response/outcome
		For patients with triple negative MBC:
		•Duration with metastatic disease (OS), >5 years, OR
		•Duration on any one therapy (PFS), >2 years, OR
		•Any exceptional response to therapy (complete or near complete response), as determined by the investigators after review of the answers to the screening questions, OR
		•Any other clinical scenario that the investigators believe constitutes an extraordinary response/outcome
Wagle *et al*. (2014)	“Exquisite sensitivity to everolimus”^6^	“Near-complete response that lasted for 18 months”
Imielinski *et al*. (2014)	“Sustained outlier response”^7^	“Near-complete clinical and radiographic remission for 5 years”; this patient was one of nine responders among 306 evaluable patients in a clinical trial
Levin *et al*. (2015)	“Exceptional responders” are those with a “highly durable (≥5 years) or ongoing clinical response”^42^	•Highly durable (≥5 years) or ongoing clinical response
		•Does not capture rapid progressors
		•Only pertains to the chemotherapy under study (capecitabine)
Van Allen *et al*. (2014)	“Near-complete histologic response”^43^	“Without recurrence more than 2 years after therapy”

Published studies were included only if they also described a “normal” response for comparison to the atypical response. This table is intended to be a representative presentation of atypical response studies and initiatives. The studies cited are not limited to breast cancer

To complete this emerging picture of patients with atypical responses, we propose the following framework to describe three distinct subgroups of patients: (1) exceptional responder: a patient with an unusually favorable response to a specific treatment protocol compared to other similarly treated patients; (2) rapid progressor: a patient with an unusually poor or no response to a specific treatment protocol compared to other similarly treated patients; and (3) exceptional survivor: a patient who has far outlived the prognosis for his or her cancer subtype and stage of disease, irrespective of whether the patient exhibited an atypical response to specific therapy(ies). Patients exhibiting an atypical response as per these three categories may have metastatic cancer or possibly another life-limiting disease.

Exceptional responses may be quantitative (i.e., tumor shrinkage, absence of new metastases) or related to duration of response. Mechanisms of rapid progression may include intrinsic or acquired resistance.^6, 8^ In addition to typical responses, the MBC Project is explicitly studying atypical quantitative responses, atypical duration of response, atypical resistance, and long-term survival. Clear categorization of subgroups of atypical responders is needed to allow prospective selection of patients for hypothesis testing and to allow comparison of results across studies. Once the response of the patients being studied is more clearly stated, researchers can then determine why the response occurs. These categories will also improve the potential for data sharing and expedite research, and can be adapted as needed when considering different clinical contexts or disease subtypes. Patients on conventional therapy as well as those in clinical trials should be included when studying atypical responses, because a community-based population will generally be more heterogeneous than a population enrolled in a trial.

### Tumor-specific molecular aberrations

Analysis of molecular aberrations, which may include mutations, translocations, duplications, fusions, truncations, and other changes, in a patient’s tumor often allows identification of the biological mechanism of a response to therapy, including an exceptionally favorable or poor response.^3, 5–7, 13^ Although genomic factors are often clearly important, a genomic explanation for an atypical response is not always identified.^14^

### Moving beyond analysis of molecular aberrations in tumors

Analysis of molecular aberrations in tumors is informative, may improve selection of therapy for certain patients, and may ultimately identify the reasons for an atypical response. However, other factors also play a role in response to therapy and should be examined in both normally and atypically responding patients. Atypical responses may occur for multiple reasons including host factors, environmental factors, tumor microenvironment, use of complementary and integrative medicine (CIM), patient co-morbidities, and the interplay among these components. The studies below provide sufficiently intriguing preliminary results that warrant further study in both normally and atypically responding patients, a necessary step toward adopting these practices into the standard of care.

Response to therapy is impacted by the biology of the tumor and the environment in which the tumor is located (microenvironment). Tumor cells may interact with surrounding vascular, immune, and stromal cells as well as hormones, secreted growth factors, cytokines, and chemokines.^15–17^ These factors are dynamic and likely contribute to tumor behavior and response or resistance to therapy.^17, 18^ Indeed, therapies such as sorafenib, sunitinib, imatinib, and bevacizumab are aimed in part at modulating these tumor microenvironment factors and present opportunities for further investigation.^19^

Co-morbidities and the drugs that patients take for them may impact atypical responses and survival in cancer patients. Cardiovascular co-morbidities reduce survival time in patients with ovarian cancer.^20^ Other studies have shown variable impacts of cardiovascular,^21^ autoimmune,^22^ and diabetic^23^ co-morbidities on patient outcomes. Certain diseases or conditions may disqualify patients from taking specific cancer-related drugs. Furthermore, development of treatment-related co-morbidities such as cardiovascular problems induced by anthracyclines and trastuzumab may preclude patients from taking the drugs that may be most beneficial.^24^ These complex situations warrant further studies relative to atypical responses.

Lifestyle factors including diet, physical activity, body mass index, smoking, alcohol consumption, and social factors may influence response to therapy and survival. Investigation of whether lifestyle factors specifically impact the efficacy of a particular cancer treatment has received little attention,^25, 26^ although initial studies are promising. Exercise^27^ or a combination of a healthy diet and physical activity^28^ are associated with improved survival after a breast cancer diagnosis. Exceptional survivors cite social factors such as good communication with their medical team, strong family support, learning about their disease, and a positive, proactive attitude as contributing factors to their unexpected longevity.^29–31^

Further investigation of the impact of co-morbidities and lifestyle factors on response to therapy, especially in patients with metastatic disease, will clarify the promising studies above. Because such data can be obtained from cost-effective patient questionnaires, prospective clinical trials should be designed to capture this information. Such questionnaires should be standardized across trials and should collect information regarding all supplements consumed, CIM therapies practiced by the patient, and co-morbidities and drugs taken for them. In addition, published studies suggest that factors such as consumption of low-dose aspirin^32^ and supplements such as Vitamin D^33^ are associated with increased survival following a breast cancer diagnosis. Due to their potential, these factors should also be queried. Analysis of these data may result in hypothesis-generating patterns that could be tested in a systematic manner. An important aspect of data acquisition is standardization of how data are collected, de-identified, and stored on a secure, accessible, and minable platform.

CIM, which includes various practices such as yoga, meditation, acupuncture, and many others, may be used in conjunction with standard of care for cancer (chemotherapy, radiation, surgery, etc.). CIM modalities are practiced by most North American women with breast cancer to manage symptoms and side effects and improve quality of life.^34–36^ Investigations are underway to elucidate additional roles for CIM such as its impact on survival and other biological parameters. An example is a randomized trial that demonstrated that mindful awareness practices reduce both stress and pro-inflammatory gene expression in young breast cancer patients who completed cancer treatment.^37^

Although some funding has been available to investigate the effects of CIM on quality of life, capital for the far more expensive assays (e.g., blood tests, etc.) needed to test the impact of CIM, supplements, and other non-regulated therapies on biological outcomes is scarce. For these promising modalities to become part of the standard of care, considerable funding for a stepwise process leading to large-scale studies in both normal and atypical responders is required.

Germline genetic polymorphisms in normal, non-tumor tissue affect the pharmacokinetics of drugs in an individual patient, contribute to responses to therapy, and may play a role in an atypical response. The cytochrome P450 family of liver enzymes metabolizes many drugs. CYP genes are highly polymorphic, resulting in various metabolism phenotypes. Multiple clinical trials are examining the contribution of various CYP genotypes to response to therapy. Drugs metabolized by the same cytochrome P450 enzyme can counteract one another,^38^ and hence, treatment with CYP inhibitors is sometimes an exclusion criterion in trial design. Some natural products consumed as CIM inhibit or activate different cytochrome P450 enzymes.^39^ Although tests are available to determine the cytochrome P450 genotype and predicted phenotype, determining the pharmacokinetics profile of an individual patient and predicting a response to therapy can be extremely complex and is not yet incorporated into the standard of care. Another example is dihydropyrimidine dehydrogenase deficiency. About 5% of individuals are deficient in this enzyme due to a mutation in its gene, leading to insufficient breakdown of drugs such as capecitabine and subsequent severe toxicity.^40^ More studies are needed to precisely predict a response to therapy by leveraging pharmacogenomics.

## Summary

Although some investigations have been launched in the US and Europe to study treatment-related responses and atypical responses in cancer patients, they have significant limitations. Some potential limitations of current investigations are that tumors are heterogeneous, the complete tumor (and other tumors within the patient) is not assessed, the tumor’s microenvironment may be excluded, and the social/lifestyle determinants that may influence the response are not considered. Thus, we suggest a blueprint to increase researchers’ knowledge regarding why some patients experience an atypical response. Due to a dearth of robust studies, we do not currently know which factors will be more important and which will be less so. Investigations have provided clues regarding factors that may be important (e.g., aspirin,^32^ Vitamin D)^33^ and warrant further examination. Questionnaires designed to capture retrospective promising information such as diet, exercise, psychosocial factors, supplements, CIM, co-morbidities, etc. may also provide important clues. Clinical trials should be carefully designed to collect de-identified (“masked”) data in a standardized manner, involve a more thorough, multi-faceted study of the whole patient, and consider ethnic, racial, lifestyle, and social differences.^41^ Routine reporting of de-identified individual patient data, and not only population means, will allow identification of atypical responders and may lead to enhanced understanding of atypical responders for future studies. The interaction among factors will be complex, and patterns may emerge with examination of normal and atypical responders participating in clinical trials or using standard therapies. A stepwise process beginning with novel and prior observations followed by rigorous testing in clinical trials could be practice-changing.

### Call to action

The MBC Alliance calls for the research community to:

1. Develop unified categories of exceptional responders, rapid progressors, and exceptional survivors, including qualitative and quantitative criteria (Table 2).

**Table 2:** Framework for three categories of atypical responders

	Response to therapy	Duration of survival
Exceptional responder	A patient who has responded unusually favorably (dramatic tumor shrinkage, development of no evidence of disease, or an unusually long progression-free survival) to a particular therapy compared with others on the same therapy who have the same cancer stage and subtype of disease (and possibly a similar number of prior lines of therapy)	The patient may or may not have survived well past his or her prognosis.
Rapid progressor	A patient who has responded unusually poorly (dramatic tumor growth or an unusually short progression-free or overall survival) to a particular therapy compared with others on the same therapy who have the same cancer stage and subtype of disease (and possibly a similar number of prior lines of therapy)	
Exceptional survivor	The patient may or may not have exhibited an atypical response to a specific therapeutic regimen.	A patient who has far outlived the prognosis for his or her cancer subtype and stage of disease.

Quantitative metrics should be developed by the research community and stated in each published study. Metrics may include standard deviation, percentile, etc. Quantitative criteria may need to be considered in terms of the clinical context including cancer type or subtype, patient population, and therapeutic regimen
2. Enhance clinical trial design to:
  - study exceptional responders and rapid progressors relative to specific treatment outcomes;
  - obtain data about multiple aspects of a response to therapy;
  - encourage collection of a common set of data elements, including health-related quality of life measures, across clinical trials;
  - use standardized questionnaires to gather more information about CIM modalities practiced, supplements consumed, co-morbidities and drugs taken for them, etc., look for correlations, and perform retrospective and prospective analyses to test hypotheses about potentially important factors.
3. Encourage funding agencies to accelerate exploration of the underlying factors governing atypical responses including CIM, co-morbidities, and the entire patient, without limiting the study to tumor markers or genetics. These factors should also be investigated in normally responding patients.
4. Investigate why all three subtypes of atypical responders show extraordinary outcomes. Exceptional survivors may not be enrolled in a trial, and stakeholders will need to work with experts to develop methodologies to study these patients and the factors contributing to their survival.
5. Standardize how data are captured and de-identified regardless of source to allow accessible mining by authorized researchers across platforms and systems. Leveraging a secure platform for access to data regarding exceptional responders, rapid progressors, and exceptional survivors will help detect survival patterns and formulate and improve testable hypotheses.

The MBC Alliance hopes progress in this area of research will glean a significantly greater understanding of patient outcomes that will be leveraged to enhance precision medicine such that patients who face an incurable cancer may, in the future, become exceptional survivors who can manage their disease as a chronic condition.
